# IL-17 deficiency aggravates the streptozotocin‐induced diabetic nephropathy through the reduction of autophagosome formation in mice

**DOI:** 10.1186/s10020-021-00285-4

**Published:** 2021-03-10

**Authors:** Kyung-Hyun Kim, Geum-Lan Hong, Da-Young Jung, Shanika Karunasagara, Won-Il Jeong, Ju-Young Jung

**Affiliations:** 1 Department of Veterinary Medicine, Institute of Veterinary Science, Chungnam National University, 99, Daehak-ro, Yuseong-gu, Daejeon, 34134 Republic of Korea; 2 Laboratory of Liver Research, Graduate School of Medical Science and Engineering, Korea Advanced Institute of Science and Technology, Daejeon, 34141 Republic of Korea

**Keywords:** IL-17A, Diabetic nephropathy, Autophagy, Autophagosome formation

## Abstract

**Background:**

Diabetic nephropathy (DN) is one of the most important medical complications of diabetes mellitus. Autophagy is an important mediator of pathological response and plays a critical role in inflammation during the progression of diabetic nephropathy. Interleukin (IL)-17A favorably modulates inflammatory disorders including DN. In this study, we examined whether IL-17A deficiency affected the autophagy process in the kidneys of mice with streptozotocin (STZ)-induced DN.

**Methods:**

The autophagic response of IL-17A to STZ-induced nephrotoxicity was evaluated by analyzing STZ-induced functional and histological renal injury in IL-17A knockout (KO) mice.

**Results:**

IL-17A KO STZ-treated mice developed more severe nephropathy than STZ-treated wild-type (WT) mice, with increased glomerular damage and renal interstitial fibrosis at 12 weeks. IL-17A deficiency also increased the up-regulation of proinflammatory cytokines and fibrotic gene expression after STZ treatment. Meanwhile, autophagy-associated proteins were induced in STZ-treated WT mice. However, IL-17A KO STZ-treated mice displayed a significant decrease in protein expression. Especially, the levels of LC3 and ATG7, which play crucial roles in autophagosome formation, were notably decreased in the IL-17A KO STZ-treated mice compared with their WT counterparts.

**Conclusions:**

IL-17 deficiency aggravates of STZ-induced DN via attenuation of autophagic response. Our study demonstrated that IL-17A mediates STZ-induced renal damage and represents a potential therapeutic target in DN.

## Introduction

The increasing prevalence of diabetes mellitus has become one of the most serious public health challenges globally. Diabetic nephropathy (DN) is one of the devastating complications of diabetes and currently the single leading cause of end-stage renal failure worldwide. The pathogenesis of DN involves hyperglycemia, which induces oxidative stress in the cells, which triggers renal inflammatory response. Various cytokines in the interleukin (IL) family modulate and mediate inflammation during the development of DN. IL-17A, a member of the IL-17 family, is produced by a subset of T helper (Th) cells, termed Th17 cells. It is a pleiotropic cytokine involved in tissue inflammation. It induces the expression of proinflammatory cytokines, chemokines and matrix metalloproteinases (MMPs). The pathogenicity of IL-17 has been implicated in rheumatoid arthritis (Lubberts et al. 2002), multiple sclerosis (Lock et al. 2002), cancer (Hyun et al. 2012) and diabetes (Emamaullee et al. 2009). However, the pathological role of IL-17 in various diseases has been disputed in several studies (Haak et al. 2009; Nakashima et al. 2012; Taleb et al. 2009).

Macrophagy (hereafter referred to as autophagy) acts as a survival mechanism under stress responses that participates in maintaining cellular homeostasis (Kroemer et al. 2010; Mizushima 2009). Autophagy begins with the formation of double-membraned autophagic vesicles (autophagosome), which subsequently enclose misfolded proteins, protein aggregates and damaged organelles. The autophagosome then fuses with lysosomes to deliver, the cargo for degradation and recycling (Mizushima 2007; Levine and Kroemer 2008). Autophagy has been widely implicated in the pathogenesis of neurodegenerative disease, carcinoma, as well as the kidney disease (Choi et al. 2013; Lenoir et al. 2016; Cybulsky 2017). Dysregulation of the autophagic pathway has been well-documented in acute kidney injury and DN (Kaushal and Shah 2016; Tagawa et al. 2016). Although the specific role of autophagy in DN remains to be elucidated, emerging evidence suggests that autophagy is deteriorated in glomerular and tubular cells in diabetes types 1 and 2. During the development of DN, the autophagic response is increased by modulating inflammatory cytokines and fibrotic changes. In this regard, the specific effects of IL-17A in autophagic response on the pathogenesis of diabetes have yet to be elucidated. Therefore, we investigated examined the role of IL-17A in autophagic response of mice with STZ-induced diabetic nephropathy and genetically deficient in IL-17A.

## Materials and methods

### IL-17A knockout (KO) animals and treatment

C57BL/6 IL-17A KO mice were kindly donated by Dr. Won-Il Jeong (Korea Research Institute of Bioscience and Biotechnology, Daejeon, Korea). Non-transgenic age-matched littermate male mice (Orient Bio, Gyeonggi-do, Korea) were used as controls. The mice were acclimated under a 12 h light/dark cycle at 23 ℃ ± 2 ℃ for 2 weeks in a specific pathogen-free animal facility at Chungnam National University with standard diet and water provided ad libitum. Male mice aged 8 weeks were used in all experiments. All animal experimental protocols were approved by the Institutional Animal Care and Use Committee of the Chungnam National University.

### DN model construction

Male WT and IL-17A KO mice were fasted for 4 h before the intraperitoneal administration of streptozotocin (STZ, Sigma-Aldrich, St. Louis, MO, USA) at a dose of 50 mg/kg (in citrate buffer) for 5 consecutive days. Citrate buffer was administered to the control animals, which were otherwise subjected to the same treatment as the diabetic animals. One week after the injections, blood glucose levels were measured to confirm the induction of diabetes. The animals were randomly assigned to four groups of six mice each: (i) wild-type mice without STZ treatment (WT Cont group), (ii) STZ-treated WT diabetic mice (WT STZ group), (iii) IL-17A knockout (KO) mice without STZ treatment (IL-17A KO Cont group), and (iv) STZ-treated IL-17A KO diabetic mice (IL-17A KO STZ group). The animals were euthanized 12 weeks after STZ injection and blood and tissue samples were harvested. Serum was subjected to blood urea nitrogen (BUN) analysis. One kidney was quickly removed for histopathological and immunohistochemical (IHC) studies, while the other was removed and stored at − 70 °C prior to the western blot assay.

### Histological and immunohistochemical analyses

Paraffin-embedded kidneys were cut into 5-µm sections, followed by deparaffinization and rehydration. The specimens were stained with hematoxylin and eosin (H&E) staining, periodic acid Schiff (PAS), and Sirius red. The quantitative analysis of renal damage was performed using the H&E-stained sections. The areas affected by tubular necrosis, brush border, desquamation, and cast formation were scored as follows: 0 (none), 1 (< 10%), 2 (10–25%), 3 (25–45%), 4 (45–75%), and 5 (> 75%). The IHC study was performed by retrieving the renal antigens Tris-EDTA solution. Endogenous peroxidase activity was blocked with 0.3% (v/v) hydrogen peroxidase in methanol for 15 min and non-specific binding was blocked with 1.5% normal goat serum for 1 h. The blocked specimens were incubated overnight with antibodies against kidney injury molecule (KIM)-1 (1:100; Genetex, Irvine, CA, USA), microtubule-associated protein 1A/1B-light chain 3B (LC3B) (1:100; Sigma-Aldrich) and ATG7 (1:100; Santa Cruz, Dallas, TX, USA). The sections were then washed with phosphate-buffered saline before incubation with horseradish peroxidase-conjugated anti-mouse or anti-rabbit antibody (1:200; AbFrontier, Seoul, Korea) in a humidified chamber for 1 h. A 3,3′-diaminobenzidine substrate kit (Vector Labs, Burlingame, CA, USA) was used to visualize the signals under a microscope. The slides were examined using an Eclipse 80i microscope (Nikon, Tokyo, Japan) and evaluated in 10 randomly selected fields.

### Western blot analysis

The kidney tissues and cells were lysed in RIPA buffer (Cell Signaling Technology, Danvers, MA, USA) and protease inhibitor cocktail (Roche, Mannheim, Germany) for 15 min on ice and centrifuged at 15 min at 4 °C. The supernatant was collected and used for Western blotting. The protein samples were separated by 6–12% sodium dodecyl sulfate polyacrylamide gel electrophoresis. The resolved proteins were transferred to polyvinylidene fluoride membrane with a wet transfer system (Bio-Rad, Hercules, CA, USA). The membranes were blocked with 5% (w/v) skim milk in 1× phosphate buffered saline (PBS) with 0.1% Tween-20 (PBS-T). Then, membranes were incubated overnight with antibodies against IL-17, IL-6, transforming growth factor (TGF)-β1, monocyte chemoattractant protein (MCP)-1, α-smooth muscle actin (SMA), fibronectin (1:1000, Abcam), p-signal transducer and activator transcription 3 (STAT3), MMP-9, p-mammalian target of rapamycin (mTOR), (1:1000, Cell Signaling Technology, Danvers, MA, USA), ATG7 (1:1000, Santa Cruz), LC3 (1:1000, Sigma-Aldrich), or β-actin (1:5000, Abcam). Then, the membranes were washed five times with PBS-T and incubated with horseradish peroxidase-conjugated secondary antibody (1:5000, AbFrontior, Seoul. Korea). Each protein expression were detected using an enhanced chemiluminescence detection kit (Thermo Fisher Scientific, Rockford, IL, MA, USA). The signal intensities were quantified using the Image Lab Software (Bio-Rad) or Image J software (Image J v1.46a; NIH, USA).

### Cell culture and treatment

Human cortex proximal tubular cells (HK2; KCLB, Seoul, Korea) were grown in Dulbecco’s modified Eagle’s medium (DMEM; Gibco, MA USA) supplemented with 5 mM glucose, 10% heat-inactivated fetal bovine serum, 100 U/mL penicillin (Gibco), and 100 µg/mL streptomycin (Gibco) at 37 °C in a 5% CO_2_ atmosphere. The media was changed every 48 h. siRNA was used to examine the cellular effects of IL-17 silencing in high glucose-induced HK2 cells. Cells were seeded at an equal density of 3 × 10^5^ cells/well in six-well plates. After an overnight pre-incubation, scrambled siControl or siRNA targeting human *IL-17* (Santa Cruz, CA, USA) was adjusted together with transfection reagent (Invitrogen, Carlsbad, CA, USA) as described in the manufacturer’s protocol for 36 h. To simulate high glucose levels, cells were cultured with DMEM containing either 5 (control) or 30 mM (high glucose) glucose (Sigma-Aldrich, MO, USA). After 24 h of incubation, the cells were harvested and were subjected to western blot analysis.

### Immunofluorescence

HK-2 cells were seeded on glass chamber slides (8 × 10^3^ cells/well) Cells were fixed with absolute alcohol for 10 min. Fixed cells were washed, blocked by using 3% bovine serum albumin in PBS for 30 min, and incubated 1 h with anti-LC3B antibody (1:200; Sigma-Aldrich). Cells were washed with PBS and were incubated with goat anti-rabbit Alexa Fluor 594 (Life Technologies) for 1 h at room temperature. Cell nuclei were counterstained with Vectorshield Mounting Medium for Fluorescence with DAPI (Vector Labs) and slides were examined by using an Eclipse 80i microscope (Nikon, Tokyo, Japan) and evaluated in 10 randomly selected fields.

### Statistical analysis

The data are expressed as the mean values ± SEM. Statistical analysis was performed using one-way ANOVA, with Tukey’s multiple comparison test. *P*-values less than 0.05 were considered statistically significant.

## Results

### IL-17A blockade and histological changes

**Fig. 1:**
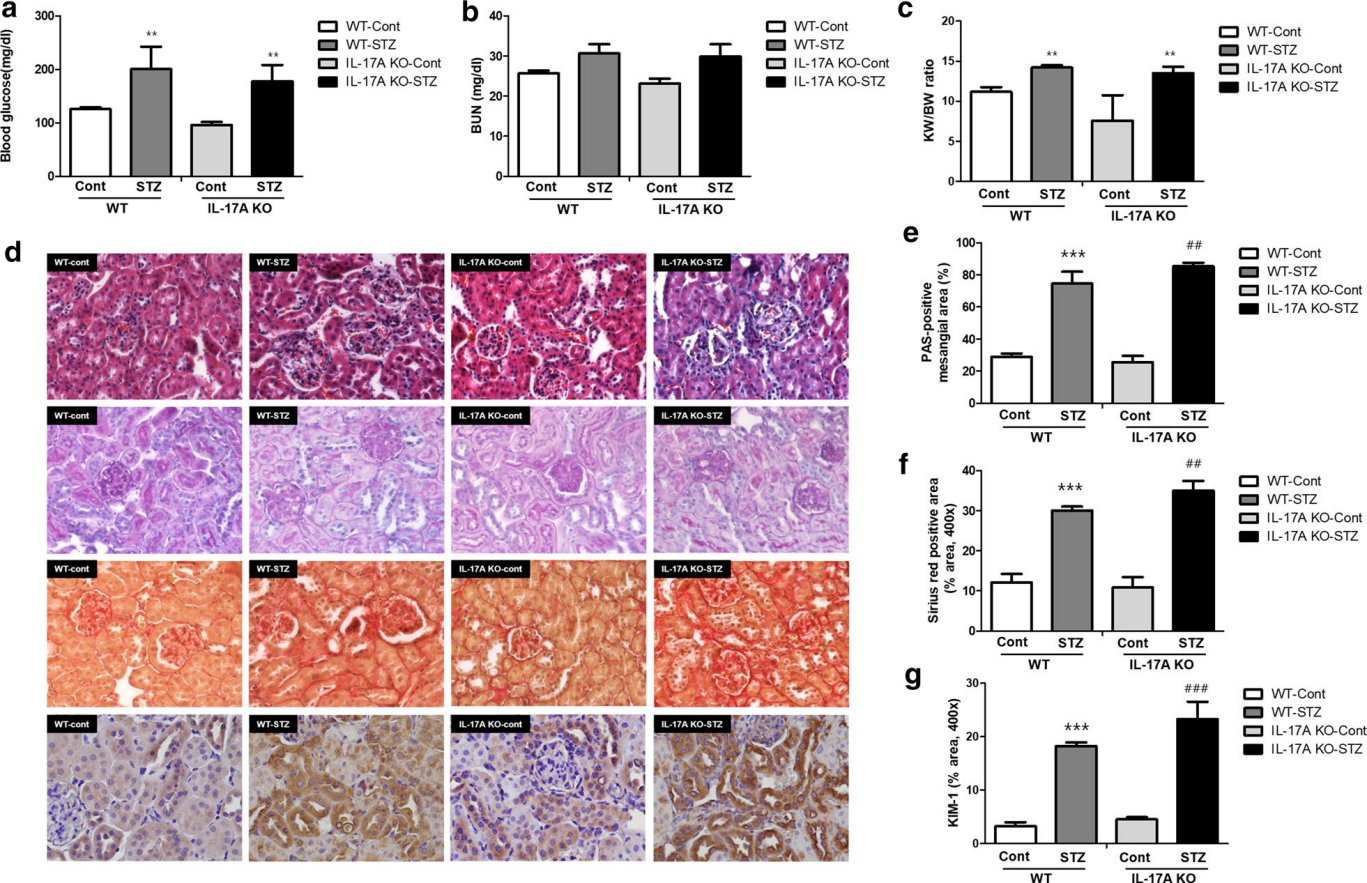
IL-17A deficiency aggravates STZ-induced DN. WT and IL-17A KO mice (n = 6/group) were injected with STZ (50 mg/mL) for 5 consecutive days (citrate buffer was administered to the control mice). The animals were euthanized 12 weeks after STZ injection. **a** Blood glucose (mg/dL), **b** serum BUN **c** kidney/body weight ratios were determined 12 weeks after STZ injection. **d** Representative histology of H&E staining, PAS staining, Sirius red staining, and immunohistochemistry for KIM-1 of the kidney. Quantification results for PAS staining (**e**), Sirius red staining (**f**), immunohistochemistry of KIM-1 (**g**). The mice were randomly assigned to four groups of six mice: (i) wild-type mice without STZ treatment (WT Cont group), (ii) STZ-treated WT diabetic mice (WT STZ group), (iii) IL-17A knockout (KO) mice without STZ treatment (IL-17A KO Cont group), and (iv) STZ-treated IL-17A KO diabetic mice (IL-17A KO STZ group). For (**a**–**c**, **e**–**g**), the data are presented as means ± SEM. ** < 0.01 and *** < 0.001 versus control group; ^##^ < 0.01 and ^###^ < 0.001 versus WT STZ group

In this study, diabetes was induced in WT and IL-17A KO mice by STZ administration. Blood glucose and kidney function were not significantly different in WT and IL-17A KO mice without diabetes. STZ treatment significantly increased blood glucose in mice of both genotypes. The WT and IL-17A KO mice displayed similar profiles in hematological and renal parameters over a 12-week period (Fig. 1a–c). Meanwhile, the H&E and PAS staining results showed increased cellular infiltration and glomerular and tubular injuries after STZ treatment of WT and IL-17A KO mice. These changes were more pronounced in the IL-17A KO mice (Fig. 1d, e). Interstitial fibrosis is a potent indicator of progression to renal failure. The Sirius red staining results showed a significant increase in interstitial fibrosis in the WT STZ group compared with the WT Cont group, but the increase was more severe in the STZ-treated IL-17A KO mice (Fig. 1d, f). In addition, IHC results for KIM-1 exhibited that STZ administration increased tubular injury and this changes more worsen in the STZ-treated IL-17A KO mice (Fig. 1d, e).

### IL-17A KO mice exhibit aggravated renal damage in STZ-induced DN

**Fig. 2:**
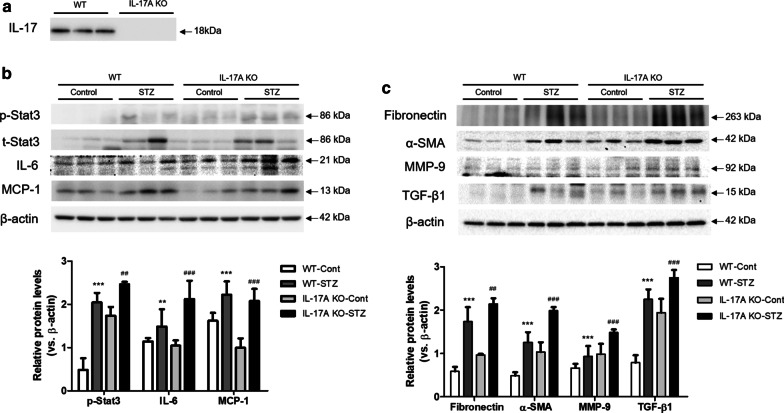
IL-17A deficiency exacerbates the expression of inflammatory cytokines and fibrosis-related proteins in STZ-induced DN. WT and IL-17A KO mice (n = 6/group) were injected with STZ (50 mg/mL) for 5 consecutive days (citrate buffer was administered to the control mice). The animals were euthanized 12 weeks after STZ injection. **a** Knockout of IL-17 was confirmed by Western blot analysis. **b** The expression of inflammatory cytokines (IL-6, p-STAT3, and MCP-1) and fibrosis-related proteins (Fibronectin, TGF-β1, α-SMA, and MMP-9) in kidney homogenates was determined by western blotting analysis. **c** The band intensities were determined by densitometry using the ratios of IL-6, p-STAT3, MCP-1, Fibronectin, TGF-β1, α-SMA, and MMP-9 to β-actin. The mice were randomly assigned to four groups of six mice: (i) wild-type mice without STZ treatment (WT Cont group), (ii) STZ-treated WT diabetic mice (WT STZ group), (iii) IL-17A knockout (KO) mice without STZ treatment (IL-17A KO Cont group), and (iv) STZ-treated IL-17A KO diabetic mice (IL-17A KO STZ group). The data are expressed as means ± SEM. ** < 0.01 and *** < 0.001 versus control group; ^##^< 0.01 and ^###^ < 0.001 versus WT STZ group

The loss of IL-17A was confirmed by immunoblotting (Fig. 2a). The role of IL-17A in DN was investigated by analyzing the expression of inflammatory cytokine and fibrosis-related genes. In both the WT and IL-17A KO mice, STZ treatment enhanced the expression of p-STAT3, IL-6, and MCP-1 compared with their control counterparts. The expression p-STAT3, IL-6, and MCP-1 was increased in STZ-treated IL-17A KO mice (Fig. 2a). Consistent with the changes in inflammatory cytokine expression, fibrosis-related proteins were up-regulated in both WT and IL-17A KO mice after STZ treatment. Especially, the increased expression of fibronectin, α-SMA, TGF-β1 and MMP-9 was observed in the STZ-treated IL-17A KO mice (Fig. 2b).

### IL-17A deficiency attenuates autophagy‐associated protein expression in STZ-induced DN

**Fig. 3:**
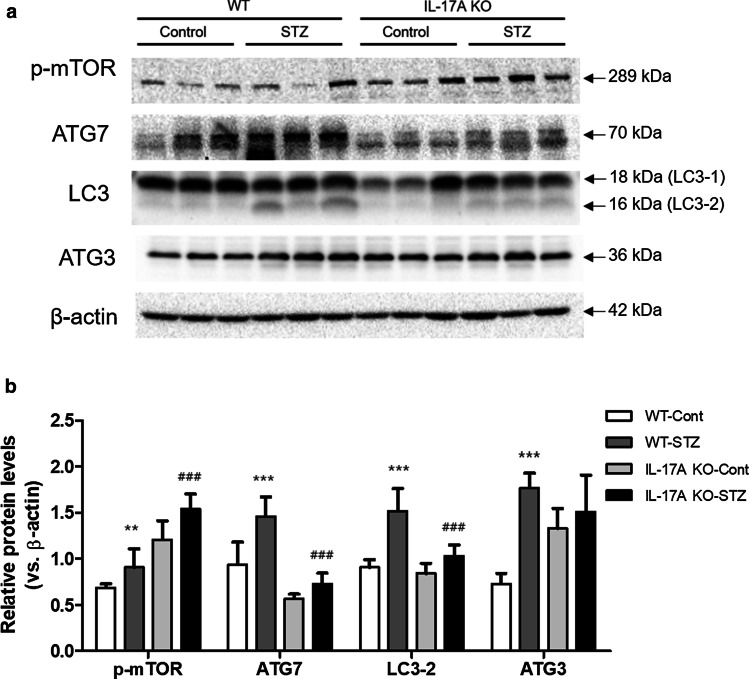
IL-17A deficiency attenuates autophagy-associated protein expression in STZ-induced DN. WT and IL-17A KO mice (n = 6/group) were injected with STZ (50 mg/mL) for 5 consecutive days (citrate buffer was administered to control mice). The animals were euthanized 12 weeks after STZ injection. **a** The expression of autophagy-associated proteins (p-mTOR, ATG7, LC3-2 and ATG 3) in kidney homogenates was determined by western blotting analysis. **b** The band intensities were determined by densitometry using the ratios of p-mTOR, ATG7, LC3-2, and ATG 3 to β-actin. The mice were randomly assigned to four groups of six mice: (i) wild-type mice without STZ treatment (WT Cont group), (ii) STZ-treated WT diabetic mice (WT STZ group), (iii) IL-17A knockout (KO) mice without STZ treatment (IL-17A KO Cont group), and (iv) STZ-treated IL-17A KO diabetic mice (IL-17A KO STZ group). The data are expressed as means ± SEM. **< 0.01 and ***< 0.001 versus control group; ## < 0.01 and ### < 0.001 versus WT STZ group

To explore the potential role of autophagy in STZ-induced IL-17A KO mice, we examined the expression of autophagy-associated protein. The autophagy process is composed of several steps, including phagophore initiation, autophagosome formation and lysosomal fusion (Mizushima 2007). The expression of mTOR, which inhibits the initial stage of autophagy, was increased by STZ treatment in both WT and IL-17A KO mice compared with the control group. However, the IL-17A KO diabetic mice showed increases in the expression of p-mTOR compared to WT diabetic mice. ATG3 and ATG7 regulate the lipidation of LC3-1 (Longatti and Tooze 2009). There was no difference in ATG3 expression between the WT and IL-17A KO mice with or without STZ treatment. Remarkably, the expression of ATG7 was increased after STZ treatment compared with WT Cont group. However, the expression of ATG7 was decreased in STZ-treated IL-17A KO mice compared with the STZ-treated WT mice. Additionally, the STZ-treated IL-17A KO mice exhibited a marked decrease in LC3-2 expression compared with the STZ-treated WT mice (Fig. 3).

**Fig. 4:**
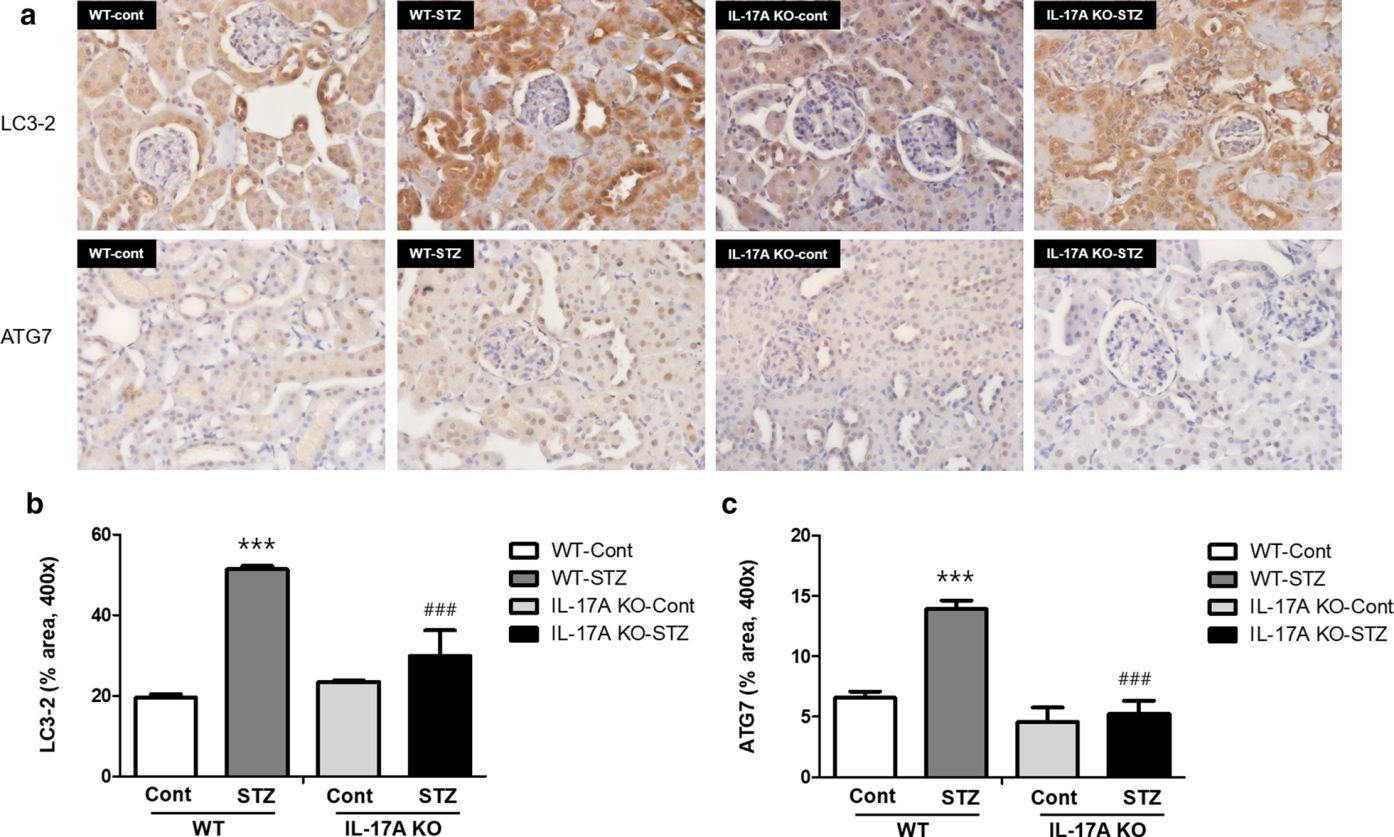
Expression of LC3-2 and ATG 7 in STZ-induced DN. WT and IL-17A KO mice (n = 6/group) were injected with STZ (50 mg/mL) for 5 consecutive days (citrate buffer was administered to control mice). The animals were euthanized 12 weeks after STZ injection. **a** Representative images of immunohistochemically stained of LC3-2 (upper) and ATG 7 (lower) of paraffin-embedded kidney sections. At 400× magnification. Quantification results for immunohistochemistry of LC3-2 (**b**) and ATG7 (**c**). The mice were randomly assigned to four groups of six mice: (i) wild-type mice without STZ treatment (WT Cont group), (ii) STZ-treated WT diabetic mice (WT STZ group), (iii) IL-17A knockout (KO) mice without STZ treatment (IL-17A KO Cont group), and (iv) STZ-treated IL-17A KO diabetic mice (IL-17A KO STZ group)

The STZ-induced alterations in the expression of autophagy-related proteins were examined in the kidneys of IL-17A KO mice via immunohistochemistry. The number of LC3B and ATG7-positive cells was increased in both STZ-treated mouse lines. Interestingly, the STZ-treated IL-17 KO mouse tissues displayed lower staining intensities for LC3B and ATG7 compared with the STZ-treated WT group (Fig. 4). These results suggest that IL-17A deficiency reduced autophagosome formation in mice with STZ-induced DN.

### IL-17A silencing enhances fibrosis-related protein expression and lowers autophagy-related protein expression in high glucose-treated HK-2 cells

**Fig. 5:**
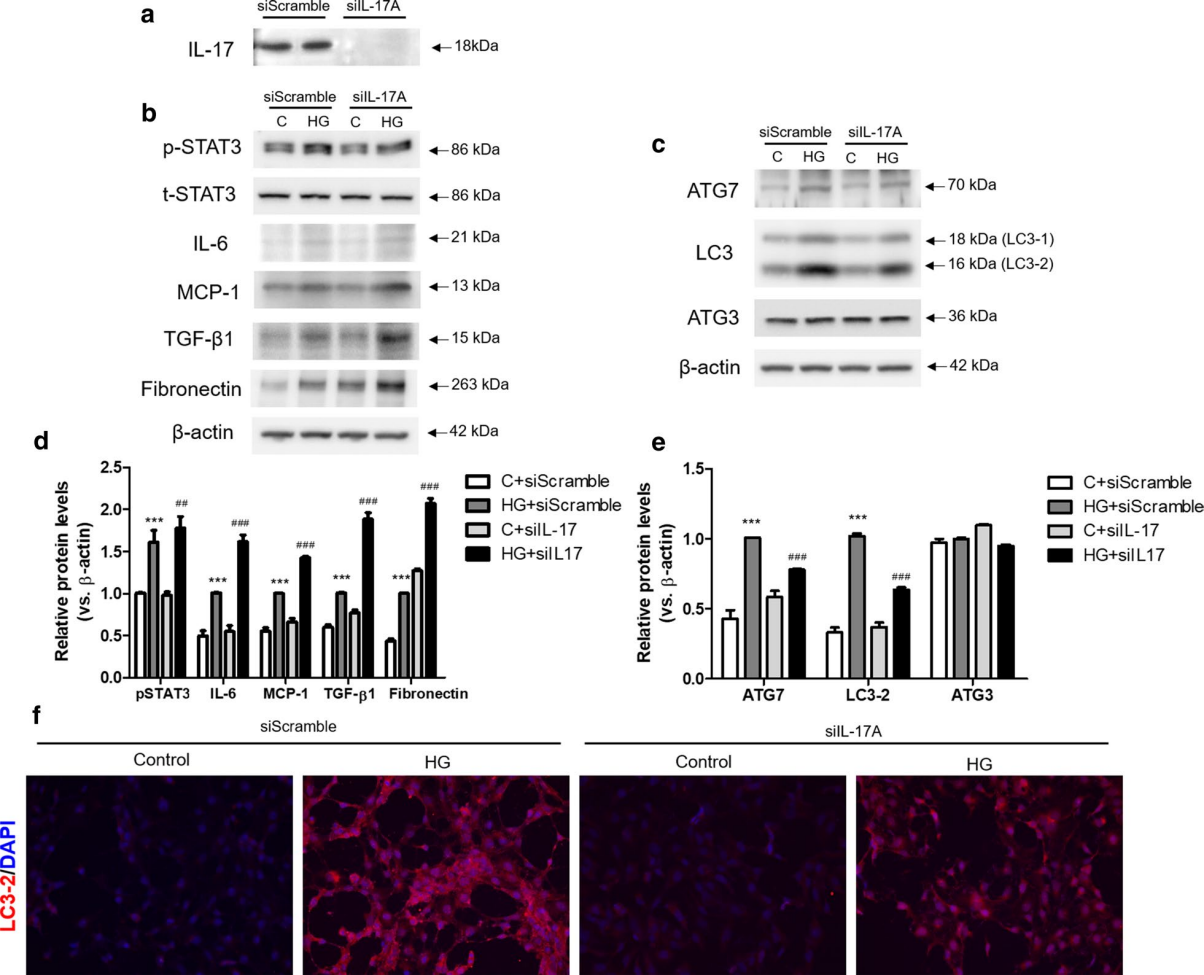
IL-17 silencing enhanced renal damages and reduced autophagy-related proteins in high glucose (HG)-treated HK-2 cells. After siScramble and si*IL-17* treatment, HK-2 cells were incubated with HG (30 mM) for 24 h. *IL-17* deletion was confirmed by Western blot analysis (**a**). The expression of inflammatory cytokines (p-STAT3, IL-6 and MCP-1) and fibrosis-related proteins (TGF-β1 and Fibronectin) in HG-treated HK-2 cells were determined by western blotting analysis (**b**) and relative band intensities subjected with β-actin (**d**). The expression of autophagy-associated protein (ATG7, LC3-2 and ATG3) were determined by western blotting analysis (**c**) and relative band intensities subjected with β-actin (**e**). Cell nuclei were visualized by 6-diamino-2-phenylindole (DAPI; blue) and LC3-2 was visualized with Alexa Fluore 594 conjugate (red) at 200× magnification (**f**). The data are expressed as means ± SEM. ** < 0.01 and *** < 0.001 versus control (**c**); ## < 0.01 and ### < 0.001 versus siScramble-transfected HG

Based on the results of STZ-induced IL-17A KO mice, we next evaluated the effect of IL-17 in high glucose (30 mM, HG)-treated HK-2 cells. To examine the effectiveness of si*IL-17* transfection, si*IL-17* treatment effectively silenced the expression of IL-17A expression in HG-treated HK-2 cells (Fig. 5a). To investigate the role of IL-17A in HG-treated HK-2 cells, the expression of inflammatory cytokines and fibrosis-related genes were analyzed. HG treatment increased the expression of p-STAT3, IL-6 and MCP-1 compared with control glucose (5 mM, C). Moreover, the expression of p-STAT3, IL-6 and MCP-1 was enhanced in HG-treated si*IL-17-*transfected cells compared HG-treated siScramble-transfected cells. Consistent with the changes in inflammatory cytokine expression, fibrosis-related proteins, TGF-β1 and fibronectin, were up-regulated in HG-treated si*IL-17-*transfected cells compared HG-treated siScramble-transfected cells (Fig. 5b, d).

To explore the potential role of autophagy in HG-treated HK-2 cells, we examined the expression of autophagy-associated protein in HK-2 cells. The expression of ATG7 and LC3-2 was increased in HG treatment. However, the expression of ATG7 and LC3-2 was decreased in HG-treated si*IL-17-*transfected cells compared HG-treated siScramble-transfected cells. There was no difference in ATG3 expression between the siScramble and si*IL-17* with or without HG treatment (Fig. 5c, e). Immunofluorescence staining results also showed that the number of siScramble-transfected cells positively stained with LC3-2 were increased after HG treatment. However, LC3-2 positive cells were reduced in HG-treated si*IL-17-*transfected cells compared HG-treated siScramble-transfected cells (Fig. 5f). Collectively, si*IL-17-*treatment enhanced the expression of renal damage and reduced the expression of autophagy-related protein expression in HG-treated HK-2 cells.

## Discussion

In this study, we demonstrated that IL-17A was a significant contributor to STZ-induced DN. The genetic deletion of IL-17A aggravated the expression of inflammatory cytokines and fibrosis-related proteins in STZ-induced DN in vivo and HG-treated HK-2 cells in vitro. These changes were attributed to the reduction of autophagy-related protein expression in IL-17A KO diabetic kidneys and HG-treated renal tubular cells.

IL-17 proteins play a positive role in regulating chronic diseases such as psoriasis vulgaris, multiple sclerosis, and rheumatoid arthritis by enhancing the induction of cytokines and extracellular proteins (Miossec and Kolls 2012; Beringer et al. 2016; Baeten et al. 2013). Although inflammation is a key modulator in the pathogenesis of DN, less is known about the specific contributions of IL-17. Recently, Mohamed et al. (2016) reported that IL-17A attenuated fibrosis and reduce tissue injuries. Several studies have suggested a paradoxical role of IL-17A in the progression of DN (Hyun et al. 2012; Galvan and Danesh 2016). However, the role of IL-17A in DN is not fully understood. Here, we provide further insights into the role of IL-17A in diabetic kidney disease.

STZ administration altered the blood glucose levels in both WT and IL-17A mice. However, interstitial fibrosis, tubular injuries, and mesangial expansion were pronounced in IL-17A KO mice than in WT mice. Similar results has been reported that IL-17A deficiency does not affect blood glucose levels and change the tubular and mesangial changes in kidney (Mohamed et al. 2016). During the pathogenesis of DN, glomerular hypertrophy; thickening of the basement, tubular, and glomerular membranes; and accumulation of ECM leads to tubulointerstitial and glomerular fibrosis and sclerosis (Kanwar et al. 2008). In this process, IL-17 positively regulates IL-6 expression, enhancing the activation of NF-κB signaling, which activates fibrotic genes including TGF-β1 and STAT3. The activation of STAT3 in renal glomerular and mesangial cells the increased the production of TGF-β1, collagen IV, and fibronectin, resulting in glomerulosclerosis in DN (Chuang and He 2010; Lu et al. 2009; Bienaime et al. 2016). The STZ-treated IL-17A KO mice in this study showed increased expressions of IL-6 and STAT3 compared with the STZ-treated WT mice. Moreover, collagen deposition and fibrosis-related proteins including fibronectin, TGF-β1, α-SMA and MMP-9 were increased in STZ-treated IL-17A KO mice compared with STZ-treated WT mice. Additionally, HG-treated renal tubular cells also showed that IL-17A silencing enhanced the expression of inflammation and fibrosis related protein compared with HG-treated siScramble-transfected cells. These results suggest that IL-17A deficiency aggravated STZ-induced renal injuries by modulating inflammation and fibrosis.

The accumulation of ECM in the glomerular and tubulointerstitial compartments is a key steps in the development of DN. Impairment in autophagic activity has been the focus of recent studies involving the pathogenesis of diabetic kidney disease. Cellular autophagy was inhibited in the renal cortex tubules of STZ-induced early diabetic rats with associated renal hypertrophy, and insulin replacement by insulin treatment or islet transplantation reversed the autophagy inhibition (Ade et al. 1992; Han et al. 1997). In addition, impaired autophagy evidenced by the renal accumulation of p62/sequestosome 1 (SQSTM1), a substrate of the autophagy-lysosomal degradation pathway, was also demonstrated in STZ-induced diabetic mice (Vallon et al. 2013) and Wistar fatty rats (Kitada et al. 2011), which are common models for type 1 and type 2 diabetes respectively. Autophagy is a self-eating catabolic pathway, which is regulated by a complex signaling network (Behrends et al. 2010). mTOR, Ser/Thr protein kinase, is a key negative regulator of the initiation of autophagy (Klionsky et al. 2016). Several studies have reported that DN pathogenesis is associated with impaired autophagic activity via activation of the mTOR pathway (Ding and Choi 2015). Human and experimental type 1 and 2 DN exhibited enhanced mTOR complex1 (mTORC1), which is composed of mTOR, the regulatory protein associated with mTOR (Raptor), and mammalian lethal with Sec13 protein (mLST8) activity (Lloberas et al. 2006; Mori et al. 2009; Godel et al. 2011). Our results also showed enhanced p-mTOR expression in STZ-induced DN mice. However, IL-17A deficiency significantly increased the expression of p-mTOR in STZ-treated mice. The expression of ATG7, which facilitates the conversion of LC3-1 to LC3-2, was increased after STZ treatment, whereas STZ-treated IL-17 KO mice showed a reduced expression of ATG7. Along with the expression of ATG7, the expression of LC3-2, which is a key marker for autophagosome formation (Choi et al. 2013), was also decreased in STZ-treated IL-17 KO mice compared with the STZ-treated WT mice. Consistent with in vivo results, the expression of ATG7 and LC3-2 was decreased in HG-treated si*IL-17-*transfected cells compared HG-treated siScramble-transfected cells. These results show that IL-17 deficiency attenuated the expression of autophagy-associated proteins in STZ-induced DN.

In diabetic kidneys, hyperglycemia results in sustained impairment of nutrient-sensing pathways and abnormal autophagy, leading to the accumulation of damaged organelles that may switch off the protective stress signals in cells reversibly. This study revealed that IL-17A deficiency exacerbated renal injury in STZ-induced diabetic mice. Consistent with our data, Krebs et al. reported that IL-17 KO mice showed aggravated renal injury in a model of deoxycorticosterone acetate salt/angiotensin II-induced hypertension. Additionally, Mohamed et al. (2016) also demonstrated that the absence of IL-17A expression increased the severity of STZ-induced diabetes. Our results provide increasing evidence supporting the role of IL-17A as a key mediator of renal damage in DN.

Impaired autophagic activity is implicated in the pathogenesis of DN. IL-17A deficiency affects the expression of ATG7 and LC3-2. The critical steps of Atg8 and Atg12 conjugation are directly involved in ATG7 which plays a role in the development of a transgenic animal model of diabetes (Jung and Lee 2009). LC3, which is a mammalian ortholog of Atg8, is essential for the conjugation of autophagosome formation, following its cleavage and lipidation into LC3-2 (Mizushima 2007). The relationship between IL-17A and autophagy has yet to be elucidated. This study demonstrates that IL-17A deficiency down-regulates the expression of ATG7 and LC3-2, which are key mediators of autophagosome formation in STZ-induced DN. Therefore, IL-17A can contribute to the renal damage during DN via regulation of autophagosome formation.

Emerging evidence suggest the role of mTORC1 signaling in the development of Th17 cells and their IL-17 expression (Nagai et al. 2013; Gulen et al. 2010). The blockade of mTORC1 signaling using rapamycin inhibits the expression of p-STAT3 and IL-17 in hepatitis B virus-infected patients. mTORC1 may drive IL-17 expression through the activation of STAT3 (Kim et al. 2014). IL-17 expression was positively regulated mTORC1 signaling via mTORC1-STAT3 signaling pathway (Ren et al. 2016). Meanwhile, mTORC1 plays critical role in the induction and regulation of autophagy in DN (Godel et al. 2011). These results support our findings of increased expression of p-mTOR in STZ-induced IL-17A deficiency. Collectively, our results suggest that the protective roles of IL-17A is associated with the regulation of autophagic response.

## Conclusions

Our findings suggest that IL-17A plays a protective role in STZ-induced diabetic kidneys via regulation of autophagic responses. These results may open new avenues for understanding the role of IL-17A in DN, and thereby provide important therapeutic options for the disease management.

## Data Availability

Not applicable.

## Abbreviations

DN: Diabetic nephropathy
IL: Interleukin
MMPs: Matrix metalloproteinases
STZ: Streptozotocin
BUN: Blood urea nitrogen
IHC: Immunohistochemical
H&E: Hematoxylin and eosin
PAS: Periodic acid Schiff
LC3: Microtubule-associated protein 1A/1B-light chain 3
TGF: Transforming growth factor-β1
SMA: α-Smooth muscle actin
STAT3: p-Signal transducer and activator transcription 3
mTOR: p-Mammalian target of rapamycin
